# Antimicrobial Resistance Profile and Associated Factors of Group B Streptococci Colonization among Pregnant Women Attending Antenatal Clinics in Jigjiga, Southeast Ethiopia

**DOI:** 10.1155/2022/9910842

**Published:** 2022-03-31

**Authors:** Addisu Tesfaye, Addisu Melese, Awoke Derbie

**Affiliations:** ^1^Department of Medical Laboratory Sciences, College of Medicine and Health Sciences, Jigjiga University, Jigjiga, Ethiopia; ^2^Department of Medical Microbiology, College of Medicine and Health Sciences, Bahir Dar University, Bahir Dar, Ethiopia; ^3^Center for Innovative Drug Development and Therapeutic Trial for Africa (CDT-Africa), Addis Ababa University, Addis Ababa, Ethiopia; ^4^Biotechnology Research Institute, Bahir Dar University, Bahir Dar, Ethiopia

## Abstract

**Background:**

*Streptococcus agalactiae* (group B *Streptococcus*, GBS) is one of the causes of maternal and neonatal morbidity and mortality in many parts of the world. It is associated with severe maternal and neonatal outcomes. The colonization rate, associated factors, and antimicrobial sensitivity (AST) profile of GBS among pregnant women in Eastern Ethiopia is less studied.

**Methods:**

A cross-sectional study was conducted from 1^st^  March to 30^th^ May, 2021 in Jigjiga. A total of 182 pregnant women with a gestational period of ≥36 weeks were included. A structured questionnaire was used to collect data on the participants' demographic and clinical history. Vaginal-rectal samples were collected by brushing the lower vagina and rectum with a sterile cotton swab for bacteriological culture. An antimicrobial sensitivity test (AST) was performed using the Kary-Bauer disk diffusion method. Data were entered and analyzed using SPSS version 25. The logistic regression model was used to find out factors associated with GBS colonization.

**Results:**

GBS colonization among pregnant women attending antenatal care was at 15.9% (29/182). The AST result showed that the majority of the isolates were sensitive to vancomycin (96.6%), chloramphenicol (96.6%), ampicillin (93.1%) azithromycin (89.7%), and penicillin (86.2%). In contrast, the isolates were found to be resistant to ceftriaxone, erythromycin, ciprofloxacin, clindamycin, and tetracycline at 17.2%, 20.7%, 27.6%, 27.6%, and 34.5%, respectively. Multidrug resistance (MDR) was noted in 4 isolates (13.79%). GBS colonization was significantly associated a with history of preterm labor (<37 weeks of gestation) (AOR = 3.87, 95% CI = 1.36–10.9) and a history of prolonged ruptured membrane (>18 hr.) (AOR = 3.44, 95% CI = 1.34–8.83).

**Conclusions:**

The colonization rate of GBS was considerably high among pregnant women attending antenatal care in the present study area. The observed antimicrobial resistance for the common drugs and the reported MDR level calls for routine screening of pregnant women for GBS and actions to minimize antimicrobial resistance (AMR) should be strengthened.

## 1. Introduction

*Streptococcus agalactiae*, also called group B *Streptococcus* (GBS), is part of the normal flora of gastrointestinal and genital tracts [1]. The bacteria use numerous adhesins and stress response apparatuses and immune evasion schemes for vaginal colonization [2]. The lower intestinal tract is the most likely reservoir of the bacteria with a secondary spread to the genitourinary system. The risk of colonization of GBS is greater among infants who are born from mother with GBS colonization. GBS transmission is vertical, transmitted throughout labor, or in utero through the transmission of the bacteria from the vaginal or the mucosa that is anorectally colonized [3].

During the time of pregnancy, the colonization of GBS could be brief, chronic, or intermittent [4]. Colonization commonly does not show any symptoms. However, at some point in pregnancy, GBS multiplication around the vaginal area can lead to maternal morbidity as likely as neonatal complications [5]. About 10 to 30% of women of childbearing age lift GBS in the recto-vaginal area without showing any symptoms. When untreated, nearly 50–75% of neonates born from a GBS infected mothers could be potentially colonized [6]. In Ethiopia, studies revealed that maternal colonization of GBS ranges from 7.2% [7] to 20.9% [3].

Different factors are associated with GBS colonization. The history of premature rupture of membranes, gastrointestinal GBS colonization, increased maternal age, low vitamin D intake, poor personal hygiene, sexual intercourse, health care occupation, and illiteracy were reported as important associated factors with GBS vaginal colonization [8–10].

GBS is one of the leading cause of infections among neonates worldwide [11–13]. In 2017, the World Health Organization (WHO) stated that infection associated with GBS caused about 147,000 infant deaths worldwide, despite the use of intrapartum antibiotic prophylaxis (IAP). With 54% of estimated cases and 65% of stillbirths and infant deaths, Africa had the highest burden [14]. While majority of GBS infections could be detected during labor or delivery, women in their post-delivery period can also be at a greater risk for invasive GBS complications even in the absence of extra risk factors [15]. The early onset of GBS infection may cause severe neurological damages and other serious neonatal outcomes [16–20].

With regard to the antimicrobial sensitivity profile of GBS, it is regarded as uniformly susceptible to penicillin, the first-line antibiotic for Intrapartum Antibiotics Prophylaxis (IAP) [11]. Macrolides (e.g., erythromycin) and lincosamides (e.g., clindamycin) are used as alternative drugs [12]. However, the global spread of antimicrobial resistance (AMR) has led to increased GBS infections among pregnant women and newborns that are difficult to treat and poses significant health concerns. Increased use of antimicrobials for prophylaxis without proper bacteriological screening is one factor that raises this concern [21]. GBS is becoming resistant to different antibiotics, including macrolides and lincosamides, and recently resistance to penicillin and fluoroquinolone was also reported [22]. A systematic review and meta-analysis conducted in China showed that significant rates of resistance to erythromycin, clindamycin, and tetracycline were observed; 0–86%, 4–84%, and 23–96%, respectively [23]. Another meta-analysis that summarized findings from 21 Africa countries also revealed that antibiotic resistance for GBS is a major concern for the whole continent. Higher antimicrobial resistance was observed against tetracycline at 82.6% and penicillin at 33.6% [24].

A previous epidemiological study conducted in two hospitals in Jigjiga, Ethiopia, discovered neonatal sepsis (24.4%) and neonatal deaths (5.7%) as a major serious issue. The study also indicates that 96% of deaths were early neonatal deaths that occurred in the first one week of life [25]. However, there is quite limited data on the colonization rate, antimicrobial susceptibility profile, and associated factors of GBS in Eastern Ethiopia and in the present study area in particular. Therefore, our study was aimed at the description of colonization, antimicrobial susceptibility profile, and associated factors of GBS among pregnant women.

## 2. Materials and Methods

### 2.1. Study Design, Area, and Period

An institution-based cross-sectional study was conducted at two selected health facilities of Jigjiga from 01 March 2021 to 30 May 2021. Jigjiga is located in the Southeastern part of Ethiopia, 621 km from the capital, Addis Ababa. The city is situated 1934 meters above the mean sea level. Based on the Ethiopian Central Statistical Agency report of the year 2015, the city had an estimated population of 304,000 of which 151,422 were men. There were two hospitals and four health centers providing delivery services in the city during the time of data collection. For this study, Jigjiga University Sultan Sheik Hassen Yabere Referral Hospital (JJUSSHYRH) and Karamara General Hospital (KGH) were selected because these hospitals had the largest client flow for antenatal care and delivery services.

### 2.2. Study Population and Sampling

Our study population were pregnant women with a gestational age of ≥36 weeks attending antenatal clinics at JJUSSHYRH and KGH during the time of data collection. The sample size was calculated based on the proportion of GBS colonization among pregnant women reported in Harar, Ethiopia, at 13.68% [26]. Using single population proportion formula, our calculated sample size was at 182. The sample size was proportionally allocated to each health facility based on their three months pregnancy visits. Accordingly, 123 and 52 participants were recruited from JJUSSHYRH and KGH, respectively.

### 2.3. Data Collection

A pretested and structured questionnaire was used to collect data on the demographic, and clinical characteristics of the study participants. Demographic data such as maternal age, residence, marital status, occupation, educational status and clinical data such as gravidity, prenatal care, urinary tract infection, outcomes of the previous delivery, prolonged rupture of membrane, and gestational age were collected.

### 2.4. Specimen Collection, Transportation, and Bacteriological Analysis

Vaginal-rectal swabs were collected during the antenatal care (ANC) follow-up by brushing the lower vagina and rectum with a sterile cotton swab by trained nurses following universal precautions. The swabs were immediately transported to the microbiology laboratory, JJUSSHYRH, within 2–4 hours using Amies transporting media maintaining the temperature at 37.

The bacteriological analysis was performed following the methods described in the center for disease control and prevention (CDC), and the American Society for Microbiology [27, 28]. Samples were inoculated on Todd Hewitt broth media supplemented with gentamycin and nalidixic acid and incubated at 37°C for 24 hours, then subcultured on a blood agar plate at 37°C for 24 hrs. Colony characteristics, Gram-stain, and catalase test were used for presumptive identification. All Gram-positive cocci, beta-hemolytic, and catalase-negative isolates were further identified by Christie–Atkins–Munch–Petersen (CAMP) and bacitracin tests. The CAMP test was used to identify CAMP-positive GBS from other beta-hemolytic Streptococci. In brief, *Staphylococcus aureus* was inoculated onto a sheep blood agar plate by making a narrow streak down the center of the plate with a loop. Then, the test organism (GBS) was streaked in a straight-line inoculum at right angles to the *S. aureus* within 2 mm. The plates were incubated at 35°C for 24°h. A positive CAMP test was indicated by an “arrowhead” shaped enhanced zone of beta-hemolysis in the area between the two cultures with the “arrow point” toward the *S. aureus* streak. No enhanced zone of beta-hemolysis was observed in a CAMP negative reaction. In addition, the bacitracin test was used to differentiate GBS from group A streptococcus which are both beta-hemolytic [29].

### 2.5. Antimicrobial SensitivityTesting (AST)

AST was performed using the Kirby-Bauer disk diffusion method. Bacterial suspension was prepared by adding 4-5 GBS colonies of the same morphology in 5 ml of sterile physiological saline equal to a 0.5 McFarland's standard that used as a reference to adjust the turbidity of the suspension. The suspension was inoculated on Mueller–Hinton agar (MHA) plates supplemented with 5% defibrinated sheep blood. Then, antibiotic disks were placed and incubated at 35–37°C overnight. GBS were tested against antibiotics that are recommended by CLSI guideline. The zone of inhibition around antibiotic disks was measured by a calibrated ruler and interpreted as sensitive, intermediate, or resistant [30].

### 2.6. Data Quality Control

Standard operating procedures (SOPs) were followed during the sample collection, transportation, and bacteriological processing steps. Standard strains: *Streptococcus agalactiae* (American Type Cell Culture (ATCC) 27956), *Staphylococcus aureus* (ATCC 24923), *Streptococcus pyogenes* (ATCC 19615), and *Escherichia coli* (ATCC 25922) were used as quality control for culture and antimicrobial sensitivity testing.

### 2.7. Data Analysis

Data were coded, cleaned, and entered into SPSS V.25 for analysis. Generated data were compiled by frequency tables and figures and other statistical summary measures. Bi-variable logistic regression analysis was carried out to identify the possible associated factors with GBS colonization. Variables with *p*-value ≤0.25 were further entered in to the multivariable logistic regression analysis. Finally, the results were presented using AOR with its 95% CI at *p*-value <0.05.

## 3. Results and Discussion

### 3.1. Characteristics of the Study Participants

A total of 182 pregnant women were included in the study. One hundred twenty-three (67.6%) of the participants were from Jigjiga University Sultan Sheik Hassan Yabere Referral Hospital and the remaining were from Karamara General Hospital. The mean age of the participants was 27.77±5.79. Most of them at 65 (35.7%), 116 (63.7%), 126 (69.2%), 83 (45.6%), and 137 (75.3%) were in the age group of 25–29 years, married, were living in an urban setting, had no formal education, and were Muslims in terms of religion (Table 1).

With regard to the reproductive history of the participants, 138 (75.8%) were multigravida, 46 (25.3%) had a history of abortion, 22 (12.1%) had a history of stillbirth, and 28 (15.4%) had a history of neonatal death. The majority of the study participants at 130 (71.4%) had antenatal visits and about 133 (73.1%) had no history of contraceptive use. Twenty-four (13.2%) of the participants had a history of preterm labor and 35 (19.2%) had prolonged membrane rupture (Table 2).

### 3.2. Group B Streptococci (GBS) Colonization

The vagino-rectal colonization of GBS among pregnant women was at 29 (15.93%). Of these, 17 (58.6%) were from JUSSHYRH and the remaining 12 (41.4%) were from KGH. There is no statistical difference (*p*-value = 0.26) on the proportion. Our finding is comparable with other studies conducted in other parts of the world; Iran (13.65%) [31], Tanzania (16.5%) [32], Cameron (14%) [33], East Africa (15.4%) [24], and Ethiopia, (13.7%) [34, 35]. However, our finding was found to be higher than studies conducted in Turkey, Iran and Italy (<10%) [36], Kenya (12%) [37], Nigeria (9.8%) [38], and Ethiopia (7.2–13.2%) [7, 39, 40]. The finding of this study was also found lower than other similar studies conducted in Saudi Arabia (27.4%) [41], Malawi (23%) [42], Kenya (25.2%) [43], Nigeria (18%) [44], Egypt (26.8%) [45], and Ethiopia (19–25%) [3, 36, 46]. The difference in the proportion of GBS colonization among different studies in Ethiopia and elsewhere in the world might be explained by the difference in geographical setting, difference in sample size, difference in personal hygiene practice, and the type of culture media used to isolate GBS.

### 3.3. Antimicrobial Susceptibility Profile of GBS Isolates

In this study, most of the GBS isolates were found to be sensitive to vancomycin and ampicillin. In contrary, they showed a moderate level of resistance for tetracycline 10 (34.5%), clindamycin 8 (27.6%), and ciprofloxacin 8 (27.6%) (Table 3). Penicillin is the first choice for IAP and ampicillin as an alternative [5]. Studies conducted around the world, including reports in Ethiopia showed that these drugs have better action against GBS than other antibiotics [3, 34, 47, 48]. In our study, the resistance of both penicillin and ampicillin was at 13.8% and 6.9%, respectively, which implies that these drugs are still good as a first choice to manage pregnant women colonized with GBS. Clindamycin and erythromycin could also be considered for patients who are allergic to penicillin once AST is performed [5]. Our finding is comparable with the studies conducted in Switzerland (8.2%) [49], Iran, Tehran (14.6%) [26], Egypt (22.6%) [50], Kenya (30.4%) [51], and Ethiopia (17.6–26.8%) [48, 52].

We reported better sensitivity of GBS for chloramphenicol and azithromycin at 96.6% and 89.2%, respectively. This result is concurring with other findings reported in Egypt [50] and similar studies in Ethiopia [52, 53]. Vancomycin was also one of the commonly utilized drugs for patients who poorly responded for clindamycin and for the cases with a risk of high anaphylaxis. In this study, GBS isolates were found with 96.6% sensitivity for vancomycin which was similar with other studies conducted in Iran [26], Brazil [54], Cameron [33], Egypt [50] ,and Ethiopia [34, 48] where GBS isolates were found 100% sensitive.

Multidrug resistance was observed in 4 isolates (13.79%) as presented in Table 4. This finding is similar with other studies in Ethiopia that reported 15.8%–43.9% MDR GBS isolates [34, 52]. In general, the high level of AMR reported in our study might be associated with different factors including but not limited to the over and misuse of drugs in the study area where there is a weak drug regulatory practice and scarce bacteriological surveillance system because of lack of routine AST testing. Most of the antimicrobials listed above are available in the local market and individuals usually use these drugs without physician prescription.

### 3.4. Factors Associated with Colonization of GBS

In the multivariable analysis, most of the variables tested in the regression model did not show statistical association with GBS colonization among pregnant women. Only two factors were found to be statistically significant; history of preterm labor (<37 weeks of gestation) (AOR = 3.87, 95% CI = 1.36–10.9, *p*-value = 0.01) and history of premature rupture of membrane (PROM) (>18 hr.) (AOR = 3.44, 95% CI = 1.34–8.83, *p*-value = 0.01). This finding is in line with other similar studies conducted in China [55], Nigeria [56], and Ethiopia [35, 48, 57]. According to different reports, GBS infection during pregnancy can cause complications like miscarriage, premature labor, or stillbirth. Likewise, PROM is also most often due to an infection in the uterus, cervix, or vagina, in this case; GBS might be an important component that should be ruled out. 

As a limitation, neonatal outcomes are not included in our report since neonates born from the study participants were not included due to constraints.

## 4. Conclusions

The colonization rate of GBS was considerably high among pregnant women attending antenatal care in the present study area. In this study, most GBS isolates were found sensitive to vancomycin, chloramphenicol, ampicillin, azithromycin, and penicillin. However, the resistance to tetracycline, clindamycin, ciprofloxacin, erythromycin, and ceftriaxone was significant. GBS colonizationwas found significantly associated with history of preterm labor and premature rupture of membrane. Therefore, stakeholders should consider routine culture-based bacteriological screening of pregnant women to help guide proper IAP. Actions to minimize AMR should be strengthened. Further large-scale studies that include neonatal outcome of GBS positive mothers should be considered.

### 4.1. Declarations

Ethical approval: Ethical clearance was obtained from Bahir Dar University College of Medicine and Health Science institutional review board (IRB). Moreover, written informed consent was obtained from all the study participants and for those participants under the age of 16 years, written informed consent was secured from their parents or guardian. Client identifiers, including names and IDs, were also kept confidential, and any other special data security requirements were maintained and assured. Bacteriological results were communicated for health professionals attending the pregnant women.

## Figures and Tables

**Table 1:** Demographic characteristics of the participants at JJUSSHYRH and KGH in Jigjiga city, Southeast Ethiopia, March–May, 2021.

Sociodemographic-characteristics			
Variable	Categories	Frequency	Percentage (%)
*Health facility*	JJUSSHYRH	123	67.60
	KGH	59	32.40

*Age group in years*	15–19	15	8.20
	20–24	39	21.40
	25–29	65	35.70
	30–34	33	18.10
	≥35	30	16.50

*Marital status*	Single	26	14.30
	Married	116	63.70
	Divorced	29	15.90
	Widowed	11	6.00

*Residence*	Urban	126	69.20
	Rural	56	30.80

*Occupation*	Civil servant	15	8.20
	Student	19	10.40
	Housewife	68	37.40
	Self-employee	22	12.10
	Daily labour	24	13.20
	Farmer	10	5.50
	Merchant	24	13.20

*Educational status*	Had no formal education	83	45.60
	Primary school	40	22.00
	Secondary school	38	20.90
	College/University	21	11.50

*Religion*	Muslim	137	75.30
	Christian	123	24.70

**Table 2:** Clinical characteristics of the participants at JJUSSHYRH and KGH in Jigjiga city, Southeast Ethiopia, March–May, 2021.

Clinical-characteristics			
Variables	Categories	Frequency	Percentage (%)
*Gravidity*	Primigravida	44	24.20
	Multigravida	138	75.80

*History of abortion*	Yes	46	25.30
	No	136	74.70

*History of stillbirth*	Yes	22	12.10
	No	160	87.90

*Antenatal care visit*	Yes	130	71.40
	No	52	28.60

*History of antimicrobial therapy*	Yes	6	3.30
	No	176	96.70

*History of contraceptive use*	Yes	49	26.90
	No	133	73.10

*HIV status*	Positive	14	7.70
	Negative	168	92.30

*Prolonged rupture of membrane >18 hr*	Yes	35	19.20
	No	147	80.80

*History of urinary tract infection*	Yes	49	26.90
	No	133	73.10

*Diabetes*	Yes	12	6.60
	No	170	93.40

*Pregnancy-induced hypertension*	Yes	6	3.30
	No	176	97.70

*History of neonatal death*	Yes	28	15.40
	No	154	84.60

*History of preterm labour (<37 weeks)*	Yes	24	13.20
	No	158	86.80

**Table 3:** Antimicrobial sensitivity profile of GBS isolated from pregnant women at JJUSSHYRH and KGH in Jigjiga city, Southeast Ethiopia, March–May, 2021.

Antimicrobial	Disk potency (*μ*g)	Sensitive *N* (%)	Intermediate *N* (%)	Resistant *N* (%)
Penicillin G	10	25 (86.2)	—	4 (13.8)
Ampicillin	10	27 (93.1)	—	2 (6.9)
Clindamycin	2	18 (62.1)	3 (10.3)	8 (27.6)
Erythromycin	15	23 (79.3)	0	6 (20.7)
Chloramphenicol	30	28 (96.6)	0	1 (3.4)
Ciprofloxacin	5	21 (72.4)	0	8 (27.6)
Ceftriaxone	30	24 (82.8)	—	5 (17.2)
Vancomycin	30	28 (96.6)	—	1 (3.4)
Azithromycin	15	26 (89.7)	0	3 (10.3)
Tetracycline	30	15 (51.7)	4 (13.8)	10 (34.5)

**Table 4:** Multidrug resistance (MDR) profile of GBS isolates from pregnant women at JJUSSHYRH and KGH in Jigjiga city, Southeast Ethiopia, March–May, 2021.

Antibiotic combination	Frequency (%)	Remark
ERY: CD	3 (10.34)	—
P: TE	1 (3.44)	—
CTX: TE	1 (3.44)	—
CD:TE	2 (6.9)	—
CD: CTX	1 (3.44)	—
P: CTX	1 (3.44)	—
ERY: CD: CIP	2 (6.9)	MDR
AMP: TE: CIP	1 (3.44)	MDR
ERY: CD: CIP:TE	1 (3.44)	MDR

^*∗*^ERY: Erythromycin CD: Clindamycin P: Penicillin TE: Tetracycline CTX: Ceftriaxone CIP: Ciprofloxacin AMP: Ampicillin.

## Data Availability

All the generated data are included in this manuscript. The original data source can be shared up on request of the principal investigator.
